# ATP and Odor Mixture Activate TRPM5-Expressing Microvillous Cells and Potentially Induce Acetylcholine Release to Enhance Supporting Cell Endocytosis in Mouse Main Olfactory Epithelium

**DOI:** 10.3389/fncel.2018.00071

**Published:** 2018-03-20

**Authors:** Ziying Fu, Tatsuya Ogura, Wangmei Luo, Weihong Lin

**Affiliations:** Department of Biological Sciences, University of Maryland, Baltimore County, Baltimore, MD, United States

**Keywords:** cholinergic modulation, purinergic receptors, xenobiotics, vesicle release, epithelial maintenance

## Abstract

The main olfactory epithelium (MOE) functions to detect odor molecules, provide an epithelial surface barrier, and remove xenobiotics from inhaled air. Mechanisms coordinating the activities of different cell types within the MOE to maintain these functions are poorly understood. Previously, we showed that superficially located microvillous cells (MCs) in the MOE expressing transient receptor potential channel M5 (TRPM5) are cholinergic and chemoresponsive and that they play an important role in maintaining odor responses and olfactory-guided behavior under challenging chemical environment. Here we investigated TRPM5-MC activation and subsequent paracrine regulation. Ca^2+^ imaging showed that TRPM5-MCs dose-dependently increase their intracellular Ca^2+^ levels in response to ATP, an important signaling molecule for airway mucociliary movement, and to an odor mixture. Pharmacological examination showed that the ATP responses are primarily mediated by P2X purinergic receptors. Interestingly, using the endocytosis dye pHrodo Red dextran, we found that chemical-activated TRPM5-MCs significantly increase the number of pHrodo-labeled puncta compared to controls without stimulation and compared to cells that do not respond to ATP or to the odor mixture. These results indicate potential vesicle recycling after release of the signaling molecule acetylcholine (ACh). Interestingly, TRPM5 knockout (KO) results in a decrease in ATP-induced pHrodo internalization. We further investigated cholinergic regulation of neighboring supporting cells (SCs). We found that ACh strongly elevates intracellular Ca^2+^ and potentiates pHrodo endocytosis in SCs. The ACh effects are diminished in the presence of atropine or M3 muscarinic receptor antagonist and in SCs lacking M3 receptors. Collectively, these data suggest that TRPM5-MCs may regulate the MOE’s multicellular network activity via cholinergic paracrine signaling for functional maintenance and adaptive plasticity.

## Introduction

Chemosensory cells expressing transient receptor potential channel M5 (TRPM5) are found in epithelia of various tissue and organs, including the respiratory, gastrointestinal, and urinary tracts. These cells detect chemical stimuli including irritants and toxicants, and play important roles in regulating respiration, protecting vital organs and epithelial defense against bacteria and parasites (Finger et al., 2003; Gulbransen et al., 2008; Lin et al., 2008b; Ogura et al., 2010; Tizzano et al., 2010; Krasteva et al., 2011; Deckmann et al., 2014; Lee et al., 2014; Gerbe et al., 2016; Howitt et al., 2016). While solitary chemosensory cells (SCCs) in the respiratory epithelium and at the entrance of the vomeronasal organ, as well as tuft cells in the gut and brush cells in the urethra, are able to relay their chemosensory activity to innervating nerves and regulate physiological activity via neural circuits (Bezençon et al., 2008; Lin et al., 2008b; Ogura et al., 2010; Krasteva et al., 2011; Deckmann et al., 2014; Saunders et al., 2014), TRPM5-expressing microvillous cells (TRPM5-MCs) in the main olfactory epithelium (MOE) are generally not innervated (Lin et al., 2008a). Actions of TRPM5-MCs following chemical stimulation, as well as mechanisms underlying their potential paracrine effects on the local multicellular epithelial network against chemical insults, are poorly understood.

The TRPM5-MCs identified in the nasal cavity reside superficially throughout the entire MOE, but not in the respiratory epithelium. Their elaborate apical microvilli extend into the mucus layer, enabling detection of xenobiotic chemicals (Hansen and Finger, 2008; Lin et al., 2008a). Using single-cell Ca^2+^ imaging, we also found that TRPM5-MCs respond to odor molecules at relatively high levels, to bacterial lysates, and to ATP with increases in intercellular Ca^2+^ levels (Ogura et al., 2011). Interestingly, TRPM5-MCs express cholinergic markers choline acetyltransferase and vesicular acetylcholine (ACh) transporter, indicating that TRPM5-MCs are capable of releasing ACh (Ogura et al., 2011). Similar findings have been found in studies of TRPM5-expressing SCCs and brush cells in the trachea and urogenital tract (Ogura et al., 2010; Krasteva et al., 2011; Deckmann et al., 2014; Saunders et al., 2014). ACh is a potent signaling molecule that can be released by neurons and non-neuronal cells to regulate a wide variety of cellular activities (Kawashima and Fujii, 2008; Wessler and Kirkpatrick, 2008). The release of ACh from urethral brush cells is further shown by Deckmann et al. (2014). There is also pharmacological evidence that SCCs release ACh as a neurotransmitter to induce nasal inflammation (Saunders et al., 2014). We further demonstrated that, in the MOE, ACh potently increases intracellular Ca^2+^ levels in supporting cells (SCs) and suppresses evoked Ca^2+^ increases in approximately half of olfactory sensory neurons (OSNs) tested (Ogura et al., 2011). Thus, it is very likely that TRPM5-MCs exert paracrine regulation to coordinate MOE multicellular activity by releasing ACh. However, the relationship between chemical-induced activation of TRPM5-MCs and ACh release has not been studied.

The MOE is made up of multiple cell types including OSNs, SCs, MCs, basal cells, and cells of Bowman’s glands and ducts (Farbman, 2000). These cells function distinctly in detecting airborne odorants with exquisite sensitivity, providing nasal epithelial lining (Gross et al., 1982) and metabolizing xenobiotics, which protects the lower airway and lungs from chemical insults (Ding and Dahl, 2003; Thiebaud et al., 2010). Because the MOE is vulnerable to random insults by airborne chemical irritants, toxicants and harmful microorganisms, mechanisms coordinating MOE to maintain its structural and functional integrity are important not only for olfaction but also for respiratory health and homeostasis because of airway continuity. Such mechanisms, which currently are poorly understood, are expected to align with activities that remove harmful chemicals in the airway.

We recently showed that TRPM5-MCs are important for maintaining olfactory function and subsequent olfactory-guided behavior in a chemically challenging environment (Lemons et al., 2017) using transcription factor Pou2f3– or Skn-1a–knockout (KO; Skn-1a^−/−^) mice lacking TRPM5-MCs (Yamaguchi et al., 2014). Under conventional housing conditions, Skn-1a^−/−^ mice exhibit normal odor-evoked electro-olfactogram responses to a panel of odorants tested, as well as normal olfactory-guided behaviors, including finding buried food and preference reactions to socially and sexually relevant odors, that are similar to those of control wild-type mice. However, when housed in a chemically challenging environment for 2 weeks, Skn-1a^−/−^ mice, but not control mice, exhibited significant reductions in odor-evoked electro-olfactograms, and their olfactory ability in guiding these behaviors is also impaired. These findings allow us to hypothesize that activated TRPM5-MCs release ACh to modulate OSNs and also to coordinate SC activity in MOE functional maintenance.

The close anatomical proximity between TRPM5-MCs and SCs enables paracrine modulation. SCs are the second most abundant cell type in the MOE (Farbman, 2000), providing a physical and chemical barrier (Rafols and Getchell, 1983), as well as structural and metabolic support for the OSNs (Getchell and Mellert, 1991; Vogalis et al., 2005). The SCs express many xenobiotic-metabolizing enzymes, which can act either intracellularly or extracellularly after being released into the mucus layer (Thornton-Manning and Dahl, 1997; Ding and Dahl, 2003; Hu et al., 2014; Asakawa et al., 2017). SCs possess numerous vesicles and vacuoles in the supranuclear regions of their cell bodies (Getchell and Mellert, 1991), presumably resulting from xenobiotic internalization for enzymatic reactions (Getchell and Getchell, 1992). However, endocytosis in SCs or vesicle recycling following secretion and mechanisms regulating the events are understudied.

In this study, we sought to further understand TRPM5-MC function and cholinergic regulation within the MOE network. Using Ca^2+^ imaging and the endocytotic dye pHrodo, we first investigated possible vesicle release by monitoring membrane or vesicle recycling in TRPM5-MCs following ATP or odor mixture-induced activation of these cells. ATP released apically from nasal epithelial cells regulates mucociliary movement for xenobiotic removal (Workman et al., 2017). ATP can also be constitutively released from the MOE (Hayoz et al., 2012) to modulate olfactory sensitivity (Hegg et al., 2003), intracellular Ca^2+^ levels in SCs (Hassenklover et al., 2008), and MOE proliferation (Kanekar et al., 2009). Second, we investigated whether ACh increases endocytosis or vesicle recycling in SCs. Finally, we probed intracellular mechanisms mediating ACh effects using pharmacological agents and muscarinic ACh receptor subtype 3 (M3-AChR)-KO mice. Our results suggest that activated TRPM5-MCs may release ACh to potentiate SC-mediated xenobiotic clearance.

## Materials and Methods

### Animals

Two- to six-month-old adult male and female C57BL/6-background transgenic and KO mice were used in this study. Both TRPM5-green fluorescent protein (GFP) and TRPM5-KO lines were originally obtained from Robert Margolskee’s laboratory. Detailed information on the generation and initial characterization of these mice is published in Clapp et al. (2006) and Damak et al. (2006). In TRPM5-GFP transgenic mice, the TRPM5-promotor drives the expression of GFP, allowing visualization of TRPM5-MCs. The endogenous gene coding for TRPM5 remains unchanged in these mice. In our initial identification of TRPM5-MCs, we had used an anti-TRPM5 antibody to immunolabel MOE sections from TRPM5-GFP mice and showed positive TRPM5 immunoreactivity in GFP-expressing MCs (Lin et al., 2008b). We generated TRPM5-KO GFP mice by cross-mating TRPM5-GFP and TRPM5-KO mice. The M3-AChR KO line was originally obtained from Jürgen Wess (Yamada et al., 2001). All animal care and use procedures were conducted in accordance with the National Institutes of Health *Guide for the Care and Use of Laboratory Animals* (2006) and approved by the Animal Care and Use Committee of the University of Maryland, Baltimore County, Baltimore, MD, USA.

### Solutions and Chemicals

For single-cell Ca^2+^ imaging and endocytotic dye imaging, Tyrode’s saline was used for the extracellular solution bathing the cells, which contained (in mM) 140 NaCl, 5 KCl, 10 HEPES, 1 MgCl_2_, 3 CaCl_2_, 10 Na-pyruvate, and 10 D-glucose (pH 7.4). Ca^2+^/Mg^2+^-free Tyrode’s saline for cell isolation was prepared by omitting MgCl_2_ and CaCl_2_ and adding 1 mM BAPTA; Ca^2+^-free Tyrode’s saline was prepared by omitting CaCl_2_. The odor mixture was prepared as stock solution containing (in mM) 19 ammonium hydroxide, 75 ethyl acetate, 83 propionic acid, and 13 triethylamine in Tyrode’s and diluted to 1:100, 1:50, 1:10 and 1:5 to determine dose-dependent responses in TRPM5-MCs. We used this mixture because our recent study indicated that TRPM5-MCs play an important role in maintaining olfactory function in mice challenged by 2-week exposure to this odor mixture (Lemons et al., 2017). Detailed justification of using these chemicals can also be found in this article. The following pharmacological agents were dissolved in DMSO and diluted into the bath solution to a final concentration, which include darifenacin (0.1 μM), pirenzepine (0.1 μM), 4-(4-Butyl-1-piperidinyl)-1-(2-methylphenyl)-1-butanone hydrochloride (AC-42, 5 μM), 1,1-Dimethyl-4-diphenylacetoxypiperidinium iodide (4-DAMP, 0.1 μM), and 2,4,6-Trimethyl-N-[3-(trifluoromethyl)phenyl]benzenesulfonamide (m-3M3FBS 25 μM). The final concentration of DMSO, which ranged from 0.01% to 0.1%, did not affect responses when applied alone. ATP, ACh, adenosine, ADP, AMP, UTP, atropine (0.5 μM), and pyridoxalphosphate-6-azophenyl-2′,4′-disulfonic acid (PPADS, 5 μM) were dissolved in Tyrode’s saline or Ca^2+^-free Tyrode’s saline. All chemicals used in this study were purchased from either Sigma-Aldrich (St. Louis, MO, USA) or Tocris (Minneapolis, MN, USA).

### Cell Isolation

The method of isolating MCs and SCs in the mouse MOE was adapted from our previous study (Ogura et al., 2011). Briefly, mice were euthanized by CO_2_ asphyxiation followed by cervical dislocation and exsanguination through an open heart. The head skin was removed, and the nose was split from the midline. Then olfactory turbinates were dissected and placed in Ca^2+^/Mg^2+^-free Tyrode’s saline containing ~2.5–4 U/ml activated papain (Worthington, Lakewood, NJ, USA) with 2 mM cysteine for 2.5–3.5 min at room temperature. Gentle pipetting at the end of enzyme incubation facilitated cell dissociation. The supernatant was transferred to an O-ring chamber on a cover slip precoated with concanavalin A (Sigma).

### Ca^2+^ Imaging

Ca^2+^ levels in isolated TRPM5-MCs and SCs were monitored as described in our previous studies (Ogura et al., 2011). Our Ca^2+^ imaging was performed in a well-ventilated room. Stimulus solutions were capped before application and were bath applied. After stimulation, the solutions were removed from the recording chamber by a vacuum pump into a sealed glass waste container. A plastic tube channeled the odorized air from the waste container to the building central exhaust system to keep the room in a low odor environment. For Ca^2+^ imaging, cells were loaded with the Ca^2+^-sensitive dye Fura-2 AM (2 μM; Molecular Probes) for 20 min. A pair of 340- and 380-nm excitation light images was captured every 3 s using an epifluorescence microscope equipped with a 40× oil objective lens (Olympus IX71), a light source/filter changer (Sutter Lambda LS), and a cooled CCD camera (Hamamatsu C9300-221). We measured Ca^2+^ levels as the ratio of fluorescence values from 340-nm and 380-nm excitation light images. We considered changes in Ca^2+^ levels as stimulus-induced responses if Ca^2+^ levels increased >2% from stable resting levels within 30 s after stimulation.

To ensure only healthy isolated cells were imaged, we checked the cell viability in three different ways. First, we examined the cell morphology; only those with a smooth appearance of the cell body (less granulated) with multiple apical microvilli were recorded. Second, we checked their resting Ca^2+^ level, since we have observed that unhealthy or damaged cells usually show a higher resting Ca^2+^ level in numerous previous Ca^2+^ imaging experiments. For the current experiment, we only recorded cells that had a resting level below 0.9 (Fura-2 340 nm/380 nm ratio). Third, we occasionally stimulated some nonresponding cells with cold saline solution (4°C), which we previously found to elicit Ca^2+^ response in TRPM5-MCs (Ogura et al., 2011).

### Endocytosis Dye Internalization Measurement

We used a dextran-conjugated water soluble dye, pHrodo Red dextran 10K (Invitrogen), to monitor endocytosis after the cells were stimulated. The dye is pH sensitive: under natural pH conditions, such as in extracellular solution, its fluorescence intensity is very weak, but when internalized into intracellular organelles with lower luminal pH, the fluorescence intensity becomes stronger. For the experiment, TRPM5-MCs isolated from TRPM5-GFP and TRPM5-KO GFP mice were first identified by their morphological features, such as apical microvilli (Lin et al., 2008a; Ogura et al., 2011) and GFP fluorescence. The cells were then loaded with the Ca^2+^-sensitive dye Fura-2 AM (see “Ca^2+^ Imaging” section) and washed. For an initial control set of images, GFP fluorescence and transluminescence light images of the cells were captured, as well as a prestimulation fluorescence image using the same optical filter setting as for later images with pHrodo dye (530–560 nm excitation, 573–647 nm emission light). Next, individual cells were stimulated with 100 μM ATP or the odor mixture for 10 s followed by a brief wash. During the stimulation, changes in Ca^2+^ levels were monitored to determine whether the cell was responsive to the stimulation. Cells were then loaded with pHrodo Red dextran 10K (25 μg/ml) for 20 min. After a brief wash, images of internalized dye were captured at 530–560 nm excitation and 573–647 nm emission light. All images were taken using an Olympus IX71 epifluorescence microscope equipped with a 40× oil UV objective lens (N.A. 1.3), a 1.6× intermediate lens, a cooled CCD camera (Hamamatsu C9300-221), and a xenon lamp with filter changer (Sutter Lambda LS) controlled by Axon imaging workbench software. The resolution of image was 7.7 pixels/μm. The number of pHrodo containing puncta in the image of TRPM5-MCs was quantified.

Similarly, we used pHrodo dye for monitoring ACh-stimulated endocytosis or vesicle recycling in SCs. Due to the intense dye labeling within the SCs, we could not distinguish individual puncta. Therefore we measured the average fluorescence intensity level of individual cell body region in the images using ImageJ software (NIH). Endocytic activity was estimated as Δ*F*/*F*_0_ = [(fluorescence level after stimulation) − (prestimulation level)]/prestimulation level.

### Immunohistochemistry

#### Tissue Preparation

Our immunolabeling procedure has been described previously (Ogura et al., 2011; Krosnowski et al., 2012; Lemons et al., 2017). Briefly, TRPM5-GFP mice were deeply anesthetized with tribromoethanol (Avertin 250 μg/g body weight), perfusion-fixed with a phosphate buffered fixative containing 3% paraformaldehyde, 19 mM L-lysine monohydrochloride and 0.23% sodium m-periodate. The nose was harvested, post-fixed for 1.5 h and then cryoprotected with phosphate buffered 25% sucrose solution overnight. The surrounding bones were manually removed following our published method (Dunston et al., 2013) and the whole MOE tissue was embedded and cut using a cryostat (Microm International, Walldorf, Germany) into 14 μm-thick sections and mounted onto charged microscope slides (Globe Scientific, Paramus, NJ, USA).

#### Immunohistochemistry

The MOE sections were rinsed and treated with Dako Target Retrieval Solution pH 9 (DAKO Cat# S2368) for 20 min at 80°C for antigen retrieval. The sections were then incubated in a blocking solution containing 2% normal donkey serum, 0.3% Triton X-100 and 1% bovine serum albumin in 0.1 M phosphate buffered saline for 1.5 h, before immunoreacted for 48 h at 4°C with the primary antibody against early endosome antigen1 (EEA1, 1:250, Sigma Cat# E4156, RRID:AB_609870). The sections were washed and reacted with a secondary antibody conjugated with Alexa Fluor 555 (1:400; Thermo Fisher Scientific Cat# A-31572, RRID:AB_162543) for 1 h at room temperature. Sections were then counterstained with DAPI and cover-slipped with Fluoromount-G (Southern Biotech). In control experiments, primary antibodies were omitted, which resulted in negative labeling. Fluorescence images were taken using an Olympus BX 61 epifluorescence microscope equipped with a spinning disc confocal unit and Slidebook 5.0 software (3i, Denver, CO, USA).

### Statistical Analysis

Data are presented as mean ± SEM. If the *F* test was not significant and homogeneity of variance was assumed, Student’s *t*-test was performed to compare results between two experimental groups. If the *F* test was significant and homogeneity of variance was not assumed, Welch’s *t*-test was used instead. The paired *t*-test was used to compare results from two experimental groups of the same cells. For comparison of data from three or more groups, we performed one-way analysis of variance (ANOVA) and Tukey’s *post hoc* multiple comparison test. To determine significant differences between percentages of cells responsive to two different stimulus conditions, Fisher’s exact test was performed using original numbers of cells observed. Prism 6.07 (GraphPad Software, San Diego, CA, USA) was used for statistical analyses. In all tests, *p* < 0.05 was considered statistically significant.

## Results

### Increase in pHrodo Internalization in TRPM5-MCs Following Activation by ATP and a Odor Mixture

**Figure 1:**
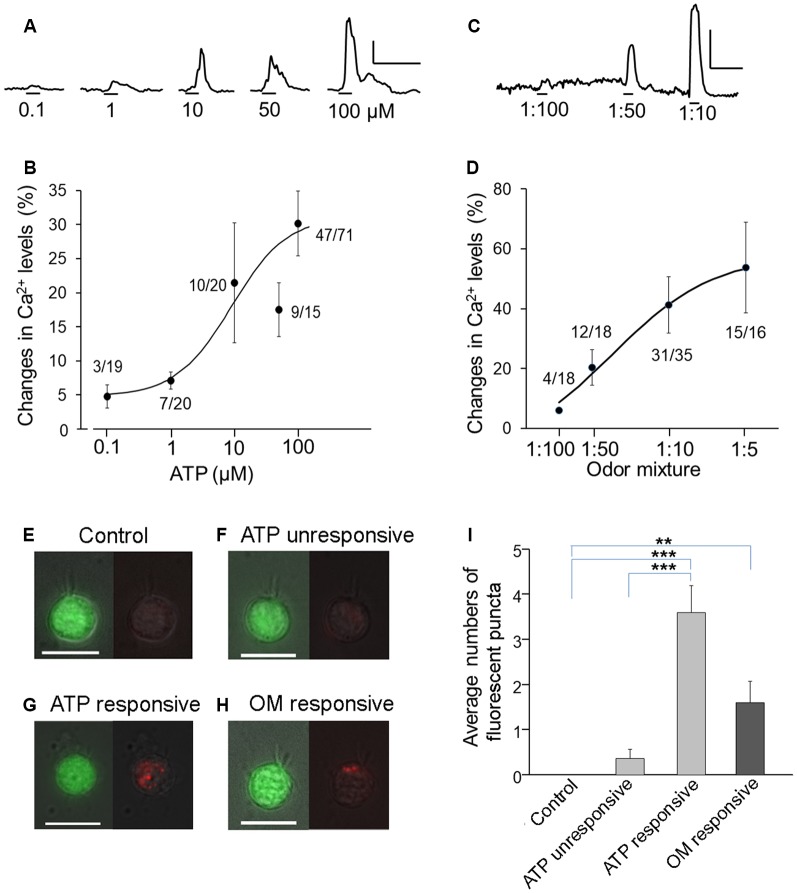
Increases in Ca^2+^ levels and endocytic dye internalization in transient receptor potential channel M5 (TRPM5)-microvillous cells (MCs) after response to ATP and odor mixture. **(A–D)** Dose responses to ATP **(A,B)** and odor mixture **(C,D)**. Traces show Ca^2+^ changes in response to different concentrations **(A,C)**; graphs show dose-peak Ca^2+^ response relations **(B,D)**. Numbers next to the plots are number of responding cells/number of cells tested. Curve fitting was calculated by the Hill equation with EC_50_ = 9 μM and Hill coefficient = 1.0 for ATP and EC_50_ = 3:100 dilution and Hill coefficient = 0.93 for odor mixture. Scale bars in **(A,C)**: 20% change from resting level and 100 ms. **(E–H)** Images of TRPM5-MCs. Representative pairs of images of green fluorescent protein (GFP; green, left panels) and internalized pH-sensitive endocytotic pHrodo dye (red, right panels) are shown for each cell. The pHrodo fluorescence images are overlaid onto a weak light image to view the cell shape. **(E)** Control cell without stimulation. **(F)** Cell that did not increase Ca^2+^ in response to 100 μM ATP. **(G)** Cell that increased Ca^2+^ in response to ATP. **(H)** Cell that responded to odor mixture (OM, 1:10 dilution). Scale bars: 10 μm. **(I)** Average number of fluorescent puncta, showing significantly increased numbers of internalized dyed puncta in ATP-responsive and odor mixture (OM)–responsive TRPM5-MCs **p < 0.01, ***p < 0.001, one-way analysis of variance (ANOVA) followed by Tukey’s multiple comparison for ATP data, and *t*-test for odor mixture data (*n* = 16–35).

Dose-dependent activation by ATP and its potential consequences in TRPM5-MCs have not been previously determined. Therefore, we stimulated freshly isolated TRPM5-MCs with various concentrations of ATP and monitored changes in intracellular Ca^2+^ using Ca^2+^ imaging. Individual TRPM5-MCs were identified by their GFP expression and by the presence of typical apical microvilli (Lin et al., 2008a) and the cell viability was examined (see “Materials and Methods” section for detail). Because the response amplitude to subsequent ATP applications was usually reduced, we applied ATP of a particular concentration only once per cell, except for low concentrations of 0.1 and 1 μM. To ensure the ATP-nonresponsive cells were viable, we randomly checked and found they could respond to cold saline solution (4°C) known to elicit Ca^2+^ response in TRPM5-MCs (Ogura et al., 2011). Figure 1A shows representative traces of ATP-induced Ca^2+^ increases in TRPM5-MCs cells, which were collected from different cells. At low concentrations, ATP-induced responses were small, and only a few cells responded (3/19 at 0.1 μM). With increasing ATP concentrations, the response amplitude generally increased with the exception of the response to 50 μM ATP. Also, the percentage of responding cells increased from 35% at 1 μM (20 cells tested) to 66% at 100 μM (71 cells tested; Figure 1B, 4–14 mice were used for each ATP concentration). These data demonstrate that both the number of responding TRPM5-MCs and their levels of intracellular Ca^2+^ increase are positively correlated to external ATP concentrations.

In our experiments, cells obtained from both males and females were used. In order to evaluate whether sex is a variable affecting our data, we alternated our daily use of animals to isolate TRPM5-MCs from either males or females and performed a set of experiments under the same experimental conditions and compared the changes in Ca^2+^ level in responses to ATP (100 μM) between males and females. We did not find a statistically significant difference in the response amplitude between males and females (*t*-test *t*_(17)_ = 0.200, *p* = 0.843). We also found a similar percentage of ATP-responding cells, which were 66% (8 out of 12 cells tested) for males and 69% (11 out of 16 cells tested) for females, respectively. Because these results did not show sex as a variable, we pooled our results obtained from both sexes.

We recently reported that 2-week exposure to a relatively strong odor mixture significantly impaired olfactory function in Skn-1a^−/−^ mice but not in control mice (Lemons et al., 2017). Those results imply that TRPM5-MCs respond to the odor mixture and subsequently modulate MOE activity for functional maintenance. We next stimulated TRPM5-GFP with the odor mixture diluted from 1:100 to 1:5 to determine dose-dependent responses using Ca^2+^ imaging. The 1:100 odor mixture elicited nearly no response (4/18 cells responded). With increasing concentrations, more TRPM5-MCs responded (12/18, 31/35 and 15/16 cells responded at 1:50, 1:10 and 1:5, respectively), and the peak amplitude of Ca^2+^ responses also increased, indicating dose-dependent activation. Unlike the response to ATP, odor mixture-induced responses were repeatable (Figure 1C: representative Ca^2+^ response traces from the same cells. Figure 1D: plot of average dose responses from responding cells. Sixteen to thirty-five cells tested from 14 mice).

TRPM5-MCs express cholinergic markers of ACh synthesis and packaging (Ogura et al., 2011). However, vesicle release of ACh and other signaling molecules from these cells has not been determined. Because the event is commonly followed by endocytotic events to recycle the membrane, we monitored the internalization of an endocytosis dye, pHrodo Red dextran. TRPM5-MCs were first loaded with Fura-2 AM to image evoked responses to either ATP or the odor mixture before pHrodo incubation. Under the control condition without stimulation, we found no dye-labeled puncta inside TRPM5-MCs imaged (Figure 1E, *n* = 7 cells). Similarly, no or very low levels of dye internalization were found in cells that did not respond to ATP (100 μM; Figure 1F, *n* = 11 cells). In contrast, ATP-responsive cells showed more pHrodo-labeled fluorescent puncta in the cytoplasm (Figure 1G, *n* = 5 cells). A similar result was obtained when TRPM5-MCs were stimulated with diluted odor mixture (1:10; Figure 1H, *n* = 10 cells). We manually counted the number of fluorescent puncta (Figure 1I, 3–6 mice for each data point). Statistical analysis indicates that significantly more fluorescence puncta were present in cells responsive to ATP than in control or nonresponsive cells (one-way ANOVA: *F*_(2,20)_ =38.49, *p* < 0.001; Tukey’s *post hoc* comparison: *p* < 0.001). Similarly, compared to controls, significantly more fluorescent puncta were found in cells that responded to the odor mixture (*t*-test: *t*_(9)_ = 3.36, *p* = 0.008). The increased internalization of the endocytotic dye pHrodo after activation suggests that TRPM5-MCs may release signaling molecules after responding to ATP or the odor mixture.

### TRPM5-MCs Respond to ATP Mainly Via Activating P2X Receptors

**Figure 2:**
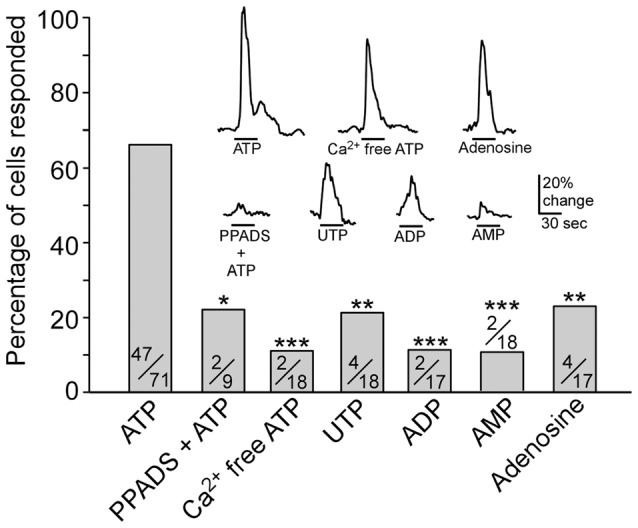
Pharmacological examination of purinergic receptors expressed in TRPM5-MCs. Plot of percentage of cells responding to each stimulus: 100 μM ATP, 100 μM ATP in the presence of 5 μM pyridoxalphosphate-6-azophenyl-2′,4′-disulfonic acid (PPADS), 100 μM ATP in Ca^2+^-free saline, 100 μM UTP, 100 μM ADP, 100 μM AMP and 100 μM adenosine. Numbers in the bars are number of responded cells/number of cells tested. *p < 0.05, **p < 0.01, ***p < 0.001, Fisher’s exact test. Inset: representative responses to each stimulus shown as changes in Ca^2+^ levels. Note: although fewer cells were responsive to Ca^2+^-free ATP and adenosine than to ATP, their response amplitudes are close to the response to ATP.

We next examined whether purinergic receptors P2X and/or P2Y were responsible for the ATP-mediated Ca^2+^ increases in TRPM5-MCs. To date, seven P2X and eight P2Y subunits are identified in other cells and the P2X subunits can form functional receptors homomerically or triheteromerically (von Kügelgen, 2006; Coddou et al., 2011). We incubated TRPM5-MCs with the nonselective P2 purinergic receptor antagonist PPADS (5 μM) for 300 s and then stimulated them with 100 μM ATP. In the presence of PPADS, approximately 22% of the recorded cells responded with very small increases in Ca^2+^ levels (Figure 2). Because P2X are ionotropic and mediate Ca^2+^ increases via Ca^2+^ influx from the external bath solution, we next monitored ATP-induced responses in Ca^2+^-free Tyrode’s solution. Approximately 11% of recorded TRPM5-MCs showed Ca^2+^ increases in response to ATP (100 μM, 2/18 cells tested), significantly lower than the 66% responding cells in normal Tyrode’s. These results suggest primary involvement of P2X subtypes (Figure 2; Fisher’s exact test: ATP vs. PPADS + ATP, *p* = 0.024; ATP vs. Ca^2+^-free ATP, *p* < 0.001, *n* = 9–71 cells from 5 to 14 mice). Because P2X subunits can form functional receptors homomerically or triheteromerically (von Kügelgen, 2006; Coddou et al., 2011), we did not pursue further pharmacological identification of specific P2X subtypes.

In cells that responded to ATP in the Ca^2+^-free condition, their response amplitude was comparable to those with external Ca^2+^ (Figure 2, inset). We therefore examined whether TRPM5-MCs express other types of purinergic receptors and the prevalence of these receptors by monitoring the percent of cells responding to the P2Y-specific ligands ADP and UTP (100 μM) and the P1 agonist adenosine. Approximately 12 and 22% of the tested cells responded to ADP and UTP (Figure 2 inset; Fisher’s exact test: ATP vs. UTP, *p* = 0.001; ATP vs. ADP, *p* < 0.001. *n* = 17–18 from four mice). These results indicate that a subset of TRPM5-MCs might express G-protein-coupled P2Y receptors. Further, we tested whether the ATP and ADP metabolite AMP could induce Ca^2+^ responses in TRPM5-MCs. We found that 100 μM AMP evoked only a small response in very few cells (2/18 cells from four mice; Figure 2 inset; Fisher’s exact test: ATP vs. AMP, *p* < 0.001). Because ectonucleotidase can convert ATP, ADP, and AMP to adenosine, we also examined adenosine (100 μM)-induced Ca^2+^ increases in TRPM5-MCs; approximately 24% of tested cells responded (4/17 from four mice), with response amplitudes comparable to those evoked by ATP (Figure 2 inset; Fisher’s exact test: ATP vs. adenosine, *p* = 0.002). This result suggests potential expression of P1 adenosine receptors in TRPM5-MCs. Taken together, our data indicate that TRPM5-MCs express multiple subtypes of purinergic receptors, with P2X subtypes being predominant.

### A Subset of TRPM5-MCs Show Spontaneous Oscillation of Ca^2+^ Levels and Elevated Endocytotic Dye Internalization

**Figure 3:**
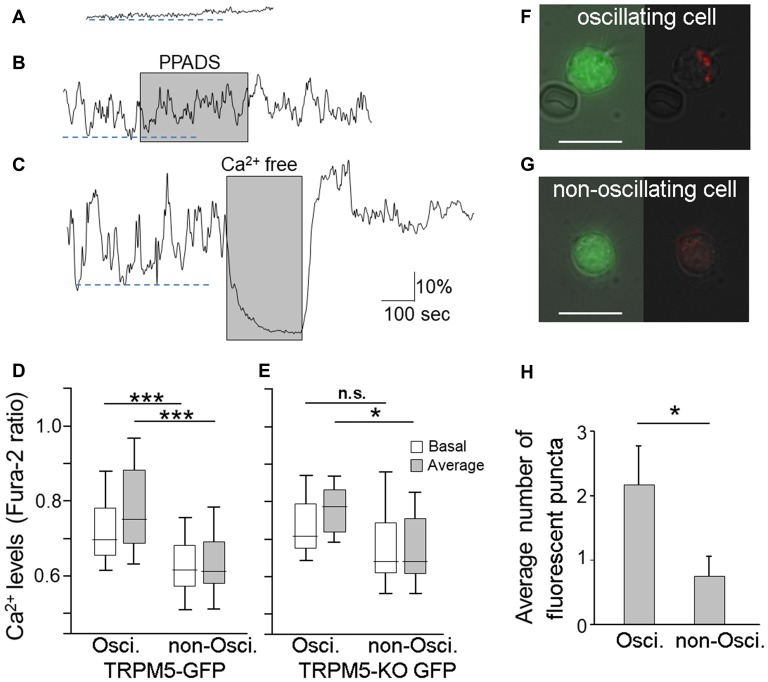
Endocytotic dye internalization in TRPM5-MCs with spontaneous Ca^2+^ oscillation.** (A–C)** Traces of changes in Ca^2+^ levels recorded from two TRPM5-MCs. One MC showed a flat baseline Ca^2+^ level throughout the entire recording time **(A)**; the other exhibited spontaneous Ca^2+^ oscillation **(B,C)**. The purinergic P2 receptor blocker PPADS (5 μM) did not suppress the spontaneous oscillation (**B**, shaded). Elimination of extracellular Ca^2+^ using Ca^2+^-free saline diminished the oscillation (**C**, shaded). Dashed lines indicate basal Ca^2+^ levels determined by averaging lower peak points of oscillation during the first 300 s in oscillating cells **(B,C)** and in non-oscillating cell **(A)**.** (D)** Basal Ca^2+^ levels and average Ca^2+^ levels from oscillating and non-oscillating cells of TRM5-GFP mice (***p < 0.001, *t*-test, *n* = 34 for oscillation cell and 75 for non-oscillating cells, respectively). **(E)** Basal Ca^2+^ levels and average Ca^2+^ levels from oscillating and non-oscillating TRPM5knockout (KO)-MCs isolated from TRPM5-KO GFP mice (*p < 0.05, n.s.: not significant, *t*-test, *n* = 8 and 8 cells for oscillation cell and for non-oscillating cells, respectively). **(F,G)** Paired images from an oscillating TRPM5-MC **(F)** show GFP (green, left panel) and internalized endocytotic dye pHrodo (red) overlaid with weak light image (right panel). A non-oscillating cell **(G)** shows no internal dye signal. Scale bars: 10 μm. **(H)** Average number of dyed puncta inside oscillating and non-oscillating TRPM5-MCs. Oscillating cells have significantly more dyed puncta than do non-oscillating cells (*p < 0.05, *t*-test, *n* = 6 for oscillation cell and 8 for non-oscillating cells, respectively).

In our Ca^2+^ imaging experiments, approximately 70% of TRPM5-MCs showed stable resting Ca^2+^ levels (Figure 3A). Our results shown in Figures 1, 2 were obtained in this set of non-oscillating TRPM5-MCs. However, the remaining 30% of the TRPM5-MCs exhibited spontaneous Ca^2+^ oscillation (Figures 3B,C). The fluctuating changes ranged between 7.9% and 100% from baseline Ca^2+^ levels (average 30.9% ± 3.8%; *n* = 34 cells), and the oscillation frequencies varied from 0.005 Hz to 0.033 Hz (0.015 ± 0.001 Hz). Application of PPADS (5 μM) had no significant effect on the oscillation amplitude or the frequency (Figure 3B, shaded), which eliminated the possible involvement of P2 receptors. The Ca^2+^ oscillation disappeared when extracellular Ca^2+^ was omitted. Interestingly, without external Ca^2+^, the baseline Ca^2+^ level was also reduced in the oscillating cells (Figure 3C, shaded). To determine whether the basal Ca^2+^ level in the oscillating cells was elevated, we compared the basal Ca^2+^ levels of both the oscillating and non-oscillating cells. We also compared the average values of Ca^2+^ levels during 300 s of recording (Figure 3D). Statistical analysis indicated that oscillating cells had significant higher basal Ca^2+^ levels and average Ca^2+^ levels than did non-oscillating cells (*t*-test: *t*_(107)_ = 4.77 and 6.24, *p* < 0.001, *n* = 34 and *n* = 75 from 14 mice, for oscillating and non-oscillating cells, respectively).

We also examined whether TRPM5 influences the oscillation using GFP-expressing MCs dissociated from TRPM5-KO GFP mice. We observed both non-oscillating and oscillating TRPM5-KO MCs. The fluctuations ranged from 7.7% to 77% (average 32.73% ± 7.67, *n* = 8) and the oscillation frequencies varied from 0.008 Hz to 0.027 Hz (average 0.015 Hz ± 0.002). These values of TRPM5-KO MCs are similar to those of TRPM5-MCs. Additionally, we compared the average values of Ca^2+^ levels TRPM5-KO MCs (Figure 3E). Statistical analysis indicated that oscillating TRPM5-KO cells had significantly higher average Ca^2+^ levels than did non-oscillating cells (*t*-test: *t*_(14)_ = 2.58, *p* = 0.022, *n* = 8 and 8 for oscillating and non-oscillating cells, respectively), but there was no significant difference in the basal Ca^2+^ levels between the two groups (*t*-test: *t*_(14)_ = 1.19, *p* = 0.254). We also examined whether there were differences in basal and average Ca^2+^ levels between TRPM5-MCs and TRPM5-KO MCs. We found no significant differences both in oscillating and non-oscillating cells (*t*_(40)_ = 0.54 and 0.26, *p* = 0.593 and 0.799, for basal and average Ca^2+^ levels in oscillating cells, and *t*_(81)_ = 1.66 and 1.23, *p* = 0.102 and 0.221, for basal and average Ca^2+^ levels in non-oscillating cells, respectively). These data indicate that TRPM5 channels do not significantly influence spontaneous Ca^2+^ oscillation in these cells.

To test whether the higher levels of intracellular Ca^2+^ in TRPM5-MCs during oscillation may cause vesicle release and subsequent membrane recycling, we incubated TRPM5-MCs with pHrodo and monitored dye internalization without stimulation (Figure 3F,G). We counted the number of dye-labeled puncta and found that oscillating TRPM5-MCs had significantly more puncta than did non-oscillating cells (Figure 3H; *t*-test: *t*_(12)_ = 2.25, *p* = 0.044; *n* = 6 and *n* = 8 for oscillating and non-oscillating cells, four and three mice, respectively). These results indicate that the oscillating TRPM5-MCs may undergo spontaneous vesicle release after elevated intracellular Ca^2+^ levels during oscillation.

### TRPM5 Knockout Results in a Decrease in ATP-Induced pHrodo Internalization

**Figure 4:**
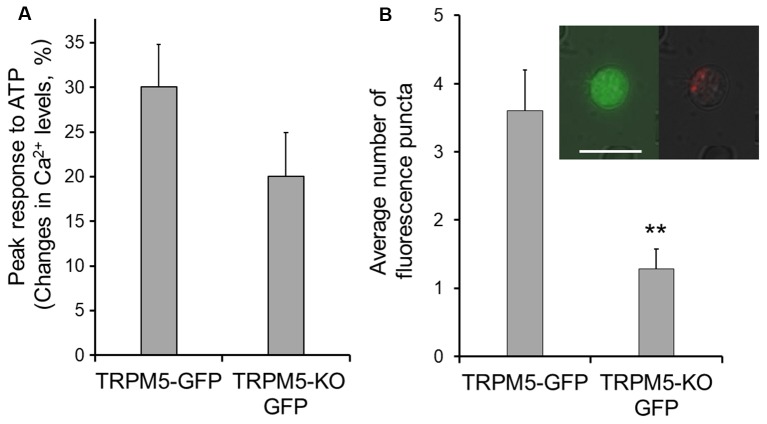
TRPM5-KO MCs of TRPM5-KO GFP mice exhibit lower endocytotic activity.** (A)** Plot of average peak Ca^2+^ changes in response to 100 μM ATP in TRPM5-MCs from TRPM5-GFP mice and TRPM5-KO MCs from TRPM5-KO GFP mice. The average amplitude trends smaller in MCs isolated from TRPM5-KO than in those from TRPM5-GFP mice, although the difference was not significant (*p* = 0.152, *t*-test, *n* = 47 and *n* = 16 for control and KO mice, respectively). **(B)** Average number of internalized pHrodo dye puncta after ATP stimulation. **p < 0.01, *t*-test (*n* = 5 and *n* = 7 ATP-responsive cells for control and KO mice, respectively). Inset: image of GFP-expressing MCs of TRPM5-KO GFP mice (green, left panel) and of the same cell showing internalized endocytotic dye pHrodo (red, right panel) overlaid with weak light image. Scale bar: 10 μm.

In the gut, TRPM5-expressing tuft cells release cytokines to initiate type 2 immunity, in which TRPM5 is indispensable (Gerbe et al., 2016; Howitt et al., 2016). We therefore tested whether TRPM5 is important for vesicle release in TRPM5-MCs of the MOE. We stimulated TRPM5-KO MCs isolated from TRPM5-KO GFP mice with ATP (100 μM) and monitored pHrodo internalization following the stimulation. Compared to the average response amplitude obtained from control TRPM5-MCs, the ATP-induced Ca^2+^ increases in TRPM5-KO MCs trended smaller, but the difference was not statistically significant (Figure 4A; *t*-test: *t*_(43.26)_ = 1.46, *p* = 0.152, *n* = 47 and *n* = 16 for control and KO, 14 and 7 mice, respectively). When examining pHrodo dye internalization following ATP stimulation, we found that ATP-responsive TRPM5 KO MCs had significantly fewer dye-labeled puncta in their cytoplasm than did TRPM5-MCs (Figure 4B and inset; *t-test:* *t*_(10)_ = 3.83, *p* = 0.003, *n* = 5 and *n* = 7 ATP-responsive cells from three control and seven TRPM5-KO GFP mice, respectively). These data imply that TRPM5 channels might enhance ATP-induced vesicle release and subsequent membrane recycling in TRPM5-MCs.

### ACh Stimulates Endocytosis in Supporting Cells (SCs)

**Figure 5:**
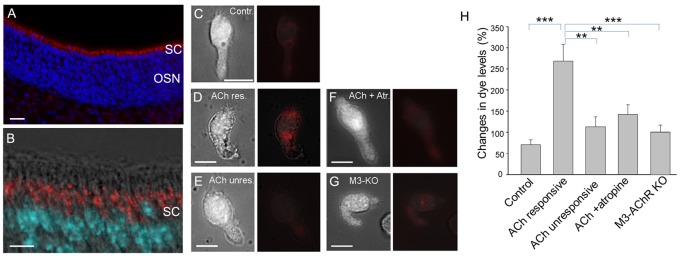
Supporting cells (SCs) of the main olfactory epithelium (MOE) take up endocytotic dye after responses to acetylcholine (ACh).** (A,B)** Confocal images of EEA1 immunolabeling (red). The MOE section was also stained with nuclear marker DAPI (blue in **A** and cyan in **B**). The higher magnification fluorescence image overlaid with a transmitted light image in **(B)** shows strong labeling of EEA1 (red) in supranuclear regions of the SCs. **(C–G)** Paired images of a SC (left panels) and the same cell showing endocytotic dye pHrodo (red) overlaid with weak light image (right panels): cell in control condition without ACh stimulation **(C)**, cell responsive to 100 μM ACh **(D)**, ACh-nonresponsive cell **(E)**, ACh stimulation in the presence of 0.5 μM atropine **(F)**, and ACh stimulation in a SC isolated from a M3-AChR-KO mouse **(G)**. Scale bars: 10 μm. **(H)** Average changes in fluorescence levels of the endocytotic dye pHrodo in the experimental conditions used for **(A–E)**. Averaged fluorescence level was measured from an entire cell body. **p < 0.01, ***p < 0.001, one-way ANOVA and Tukey’s *post hoc* test (*n* = 5–10).

We had previously hypothesized that TRPM5-MCs modulate activity of SCs by releasing ACh, based on the results showing that TRPM5-MCs are cholinergic and that ACh potently increases Ca^2+^ levels in SCs via muscarinic ACh receptors (AChRs; Ogura et al., 2011). However, it is not known whether ACh-induced increases in Ca^2+^ levels result in endocytosis in the SCs, or the underlying molecular pathways. Using an antibody against EEA1 in immunolabeling, we first showed the presence of early endosome primary in the supra-nuclear regions, indicating endocytotic activity in SCs (Figures 5A,B). We next monitored ACh-induced changes in Ca^2+^ levels and pHrodo internalization in SCs using Ca^2+^ imaging and pHrodo dye. Without ACh stimulation, small amounts of dye were internalized under control conditions, resulting in an average 71% change in fluorescence intensity (Figures 5C,H). When stimulated with 100 μM ACh, the ACh-responding SCs were strongly fluorescent, with an average 268% change in fluorescence intensity, indicating active dye internalization (Figures 5D,H). Because of the substantial amount of dye internalized, we measured the fluorescence intensity of entire SCs and the average changes in the pHrodo dye intensity. SCs that did not respond to ACh showed only low levels of labeling, slightly more than control (Figures 5E,H). Because our previous study showed that ACh-induced Ca^2+^ increases in SCs are blocked by the muscarinic AChR antagonist atropine (Ogura et al., 2011), we next tested SC endocytosis in the presence of 0.5 μM atropine, which significantly attenuated the 100 μM ACh–induced pHrodo uptake (Figures 5F,H). Furthermore, we determined the involvement of M3-AChR using SCs isolated from M3-AChR-KO mice. pHrodo dye uptake in SCs with a null M3-AChR was significantly reduced, to the level similar to that obtained from ACh-nonresponsive SCs or in the presence of atropine in control SCs with a functional M3 (Figures 5G,H). Statistical analysis showed significantly higher levels of dye intensity in ACh-responsive SCs than in those from wild-type mice without stimulation or in the presence of atropine, or in SCs from M3-AChR–KO mice (one-way ANOVA: *F*_(4,33)_ = 8.639, *p* < 0.001; Tukey’s *post hoc* comparison: *p* < 0.001, *n* = 7 and *n* = 8, for control vs. ACh-responsive; *p* = 0.005, *n* = 8 and *n* = 8 for ACh-unresponsive vs. ACh-responsive; *p* = 0.007, *n* = 10 and *n* = 8 for ACh + atropine vs. ACh-responsive; *p* < 0.001, *n* = 5 and *n* = 8 for M3-AChR KO vs. ACh-responsive, three mice were used for each data point). These data strongly suggest that ACh enhances endocytosis by activating the M3-AChR in ACh-responsive SCs.

### Supporting Cells Respond to ACh Mainly Via M3-AChR

**Figure 6:**
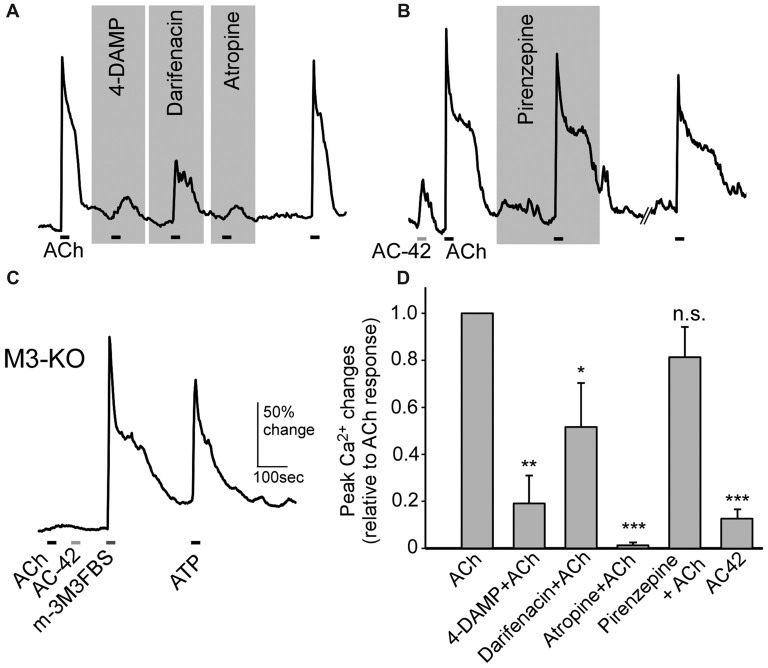
SCs of the MOE increase Ca^2+^ levels in response to ACh via M3-subtype muscarinic ACh receptors.** (A,B)** Changes in Ca^2+^ levels in response to 100 μM ACh from SCs in the presence of the M3-AChR antagonists 1,1-Dimethyl-4-diphenylacetoxypiperidiniumiodide (4-DAMP; 0.1 μM), darifenacin (0.1 μM) and the pan-muscarinic AChR antagonist atropine (0.5 μM; **A**), and in the presence of the M1 muscarinic AChR antagonist pirenzepine (0.1 μM) and in response to the M1 muscarinic AChR agonist AC-42 (5 μM; **B**). The ACh response was recoverable after wash. Horizontal bars under the traces indicate application of ACh or AC-42. **(C)** Recording of changes in Ca^2+^ levels from a SC of an M3-AChR-KO mouse. The cell failed to respond to ACh and AC-42 but responded to the phospholipase C (PLC) activator m-3M3FBS (25 μM) and to ATP (100 μM). **(D)** Average response amplitudes in the presence of the muscarinic receptor antagonists used in **(A,B)** and to the M1 muscarinic AChR agonist AC-42, as a percentage of average responses to ACh alone in the same cells. Statistical analysis was performed using the original amplitude values between ACh alone and each condition using paired *t*-tests. *p < 0.05, **p < 0.01, ***p < 0.001, n.s.: not significant (*n* = 9–11 for antagonists, *n* = 43 for AC-42).

Using subtype-specific antibodies, we previously showed strong M3- and also some M1-AChR immunoreactivity in SCs (Ogura et al., 2011). However, pharmacological studies have not been done to confirm the results, and the role of M1 has not been examined. Using Ca^2+^ imaging, we found that the ACh-induced Ca^2+^ increases were greatly reduced in the presence of the M3-AChR–selective antagonists 4-DAMP (0.1 μM) or darifenacin (0.1 μM). As control, we tested the muscarinic nonselective antagonist atropine, which produced similar suppression (0.5 μM; Figure 6A). The ACh responses were recovered after the antagonists were washed off with normal saline (Figure 6A). We next examined the effect of the M1-AChR–selective agonist AC-42 (5 μM) and found it induced a smaller response than did ACh (Figure 6B). Also, an application of ACh in the presence of the M1-AChR–selective antagonist pirenzepine (0.1 μM) resulted in only a very small reduction in the response amplitude (Figure 6B). Furthermore, in SCs isolated from M3-AChR-KO mice, both ACh and AC-42 failed to elicit responses (Figure 6C; *n* = 19 cells tested). M3-AChR-KO SCs were vital, as the same cells responded to the phospholipase C (PLC) activator m-3M3FBS (25 μM) and ATP (100 μM; Figure 6C). The data are summarized in Figure 6D, which plots the averaged ACh responses alone or in the presence of M3 and M1 antagonists, as well as responses to the M1 agonist. The ACh responses were significantly smaller in the presence of antagonists 4-DAMP (paired *t*-test: *t*_(8)_ = 4.21, *p* = 0.002, *n* = 9), darifenacin (paired *t*-test: *t*_(7)_ = 2.36, *p* = 0.025, *n* = 8), and atropine (*t*_(7)_ = 5.14, *p* = 0.001, *n* = 8) but not pirenzepine (paired *t-test:* *t*_(10)_ = 1.62, *p* = 0.069, *n* = 11). Similar statistical analysis shows that AC-42 responses are significantly smaller than ACh responses (paired *t*-test: *t*_(42)_ = 8.12, *p* < 0.001, *n* = 43). Four to 10 mice were used for each data point. Taken together, these pharmacological/physiological results strongly indicate that the M3-AChR is the main excitatory muscarinic receptor mediating ACh modulation of intracellular Ca^2+^ and endocytosis in SCs.

## Discussion

In this study we investigated chemical responses and potential vesicle release of signaling molecule ACh in chemoresponsive and cholinergic TRPM5-MCs and ACh-mediated functional modulation of SCs. Our results showed that stimulation of TRPM5-MCs with the signaling molecule ATP and the odor mixture resulted in increases in intracellular Ca^2+^ and the number of responding cells in a dose-dependent fashion, and ATP responses were primarily mediated by P2X receptors. Importantly, the activated TRPM5-MCs showed a significant increase in the number of pHrodo labeled puncta within their cell bodies, indicating membrane recycling following presumably vesicle release of ACh. TRPM5 KO results in a decrease in ATP-induced pHrodo internalization. Interestingly, spontaneous pHrodo uptake can be found in TRPM5-MCs with elevated and oscillated intracellular Ca^2+^. Furthermore, we demonstrated that ACh significantly increased intracellular Ca^2+^ levels and potentiates endocytosis in the SCs. Additionally, we provided evidence for the dominant role of the M3-subtype muscarinic receptors in mediating the ACh effects in SCs. These results are consistent with our previous findings and further demonstrate cholinergic mechanisms in regulating and coordinating MOE multicellular network.

### Purinergic Responses and Expression of Purinergic Receptors in TRPM5-MCs

ATP and its derivatives are known to potently regulate a wide variety of cellular processes in both neuronal and non-neuronal cells via their specific receptors, including exocrine and endocrine secretion, immune responses, inflammation (Burnstock, 2006). Purinergic receptors are divided into P1 type, with adenosine being the endogenous agonist, and P2 type, which can be further divided into ionotropic P2X and metabotropic G-protein coupled P2Y subtypes. The P2X receptors are non-selective cation channels in general. Activation of the P2X receptors, which we found to be the dominant receptors mediating ATP responses in TRPM5-MCs in this study, is expected to depolarize the cell membrane and increase intracellular Ca^2+^ levels via Ca^2+^ influx. The Ca^2+^ increase may be further enhanced by voltage-gated Ca^2+^ channels or other Ca^2+^-activated TRPM5 channels, which are known to potentiate the transduction signal in OSNs (López et al., 2014). TRPM5 is also an essential signaling molecule in taste and other chemosensory receptor cells (Liman, 2007). In our study, we found that ATP response in TRPM5KO-MCs trended smaller and there is a significant reduction in the number of pHrodo-labeled puncta, suggesting that TRPM5 may amplify the ATP signaling in TRPM5-MCs.

While our data obtained from experiments with the nonselective P2 receptor antagonist PPADS (Ralevic and Burnstock, 1998) and Ca^2+^-free saline demonstrate P2X being the dominant ATP receptors in TRPM5-MCs, the Ca^2+^ responses induced by UTP and ADP indicate that a small percentage of TRPM5-MCs (approximately 22% and 12%) also express P2Y-subtype receptors. Consistently, a few cells responded to ATP in Ca^2+^-free saline, with the response amplitude similar to that obtained in normal saline. This result is expected because activation of P2Y_1,2,4,6,11_ results in Ca^2+^ release from internal Ca^2+^ stores via G_q/11_ and the PLC-IP3 pathway (Fredholm et al., 2011). Such Ca^2+^ increases would persist in Ca^2+^-free saline.

Our data also indicate the potential presence of P1 receptors since adenosine increased Ca^2+^ levels in about 24% cells. The P1 receptors include A_1,2A,2B,3_, all of which are G-protein coupled receptors either stimulating (A_1_ and A_3_) or inhibiting (A_2A_ and A_2B_) adenylate cyclase activity (Fredholm et al., 2011). A_2B_ can also couple through G_q/11_ to regulate PLC activity (Burnstock, 2007). Therefore depending on P1 subtypes and their downstream signaling, adenosine-mediated purinergic modulation can be diverse. Our result that adenosine application led to an increase in Ca^2+^ level implies that TRPM5-MCs express stimulatory subtypes and/or G_q/11_ coupled type. The sources of adenosine in the MOE are not determined. Adenosine in other tissues can be generated intracellularly and transported to the extracellular matrix via transporters or extracellularly via ectonucleotidases-mediated metabolizing ATP and its derivatives. A recent study found expression of ectonucleotidases in nasal epithelial cells of zebrafish (Wakisaka et al., 2017). Currently, it is unknown whether TRPM5-MCs express ectonucleotidases on the surface of the cell membrane. In our single-cell recording condition, adenosine generated through ATP metabolism may not be potent enough to evoke responses even if the enzymes are present, due to quick perfusion of the bath solution. However, adenosine generated by surrounding cells in the nose might activate TRPM5-MCs via adenosine receptors under *in vivo* conditions.

The finding that ATP stimulates TRPM5-MC endocytosis of pHrodo, potentially following vesicle release of ACh, is significant because it may provide a mechanism connecting cholinergic and purinergic signaling for the MOE to act in a concerted fashion with the rest of the respiratory mucosa in defense against xenobiotic insults. ATP is an important signaling molecule for airway health and diseases. Nasal epithelial cells release ATP apically via pannexin channels and P2X7 channels to regulate ciliary beating frequency, which plays an important role in airway xenobiotic clearance (Workman et al., 2017). Elevated ATP release can also be caused by hypotonic stress or mechanical stimulation (Seminario-Vidal et al., 2011). ATP can also be released from the MOE in neonatal slice preparations (Hayoz et al., 2012). The released ATP is postulated to play a role in neuronal homeostasis (Jia et al., 2009), modulating olfactory sensitivity (Hegg et al., 2003), changing intracellular Ca^2+^ in SCs (Hassenklover et al., 2008), and MOE proliferation (Kanekar et al., 2009). Both P2X and P2Y receptors have been reported in OSNs and SCs based on Ca^2+^ imaging and immunohistochemistry (Hegg et al., 2003; Gayle and Burnstock, 2005; Hassenklover et al., 2008; Hayoz et al., 2012). However, expression of purinergic receptors in TRPM5-MCs has not been previously reported until this study.

In our Ca^2+^ imaging study, we found that TRPM5-MCs desensitized in response to repeat ATP stimulation, which prevented us from obtaining ATP dose responses from a single cell. Because the dose response curve was generated by recordings of different cells that were stimulated only once with a particular ATP concentration, with the exception of two lowest concentrations, the variability tended to be large. While more TRPM5-MCs responded to ATP as ATP concentration increased, the average ATP response amplitude obtained with 10 μM ATP was larger than that with 50 μM ATP although difference was not statistically significant (*p* = 0.72, *t*-test). P2X receptors desensitize and the degrees of which are subunits-dependent. It has been shown that P2X_1–2_ and P2X_2_ desensitize rapidly while (P2X_3,4,5,7_) undergo slow or no desensitization using whole-cell patch recordings from cell lines heterologously expressing homomers of the specific P2X subtypes (North, 2002; Giniatullin and Nistri, 2013; Hausmann et al., 2015). Both fast and slow or non-desensitized P2X are present in nasal epithelia (Hegg et al., 2003; Gayle and Burnstock, 2005). Molecular identity of the P2X receptors in TRPM5-MCs may provide insight into the desensitization.

### Response to Odor Mixture in TRPM5-MCs

The odor mixture used in this study was the same as that used for the 2-week exposure experiments in our previous study, in which we discovered the role of TRPM5-MCs in maintaining the olfactory responses and guided behaviors (Lemons et al., 2017). The odor components in this mixture were selected because they either are commonly used in manufacturing and regulated for occupational health or are secreted from bacteria known to be present in the nose (Kuwabara et al., 2007; Boase et al., 2013). The new data obtained directly from TRPM5-MC Ca^2+^ imaging in the present study showed that at a 1:50 dilution, which would be a concentration range between 0.3 and 1.7 mM for individual odorants, the odor mixture induced increases in Ca^2+^ levels in approximately 67% of cells, supporting our previous hypothesis that TRPM5-MCs are responsive to strong environmental odorants.

### Vesicle Release and Membrane Recycling in TRPM5-MCs

How may TRPM5-MCs exert their protective role to maintain MOE function after activation by chemical stimuli, as revealed in our recent publication (Lemons et al., 2017)? If TRPM5-MCs are to coordinate or modulate activities of the multicellular MOE, paracrine signaling via ACh may be a major mechanism since ACh has been shown to modulate activities of OSNs and SCs (Jia et al., 2009; Ogura et al., 2011). Further, chemosensory cells in the nose, trachea, gut and urethra are cholinergic (Ogura et al., 2010, 2011; Krasteva et al., 2011; Deckmann et al., 2014; Saunders et al., 2014; Schütz et al., 2015; Hayakawa et al., 2017) and ACh release from the isolated cells was measured in urethra (Deckmann et al., 2014). However, evidence of vesicle release is missing for TRPM5-MCs, as well as for other SCCs. In the gastrointestinal tract, TRPM5-expressng tuft cells release the cytokine interleukin-25 to initiate type 2 immunity against parasite infection (Howitt et al., 2016; von Moltke et al., 2016). Cytokines are commonly packaged in vesicles and released via exocytosis, which would be accompanied by membrane recycling (Stanley and Lacy, 2010). However, cholinergic brush cells in the gut and biliary tract do not express vesicular ACh transporter, which implies non-vesicle release of ACh (Schütz et al., 2015). In our study, we examined vesicle release using the endocytosis dye pHrodo. The significant increases in the number of pHrodo-labeled puncta in TRPM5-MCs following ATP- and odor mixture-induced increases in intracellular Ca^2+^ suggest that activation of these cells leads to vesicle release and subsequent recycling that internalizes the dye. Because the epi-florescence images of pHrodo-labeled puncta are convoluted and as such, they would most likely appear larger than the actual sizes.

### Role of TRPM5 in Ca^2+^ Signaling and Vesicle Release in TRPM5-MCs

In the gut, TRPM5 KO in tuft cells significantly impaired the type 2 immunity against parasite (Howitt et al., 2016), indicating that TRPM5 plays an important role in the function of these chemosensory cells. Consistently, we found that TRPM5 KO significantly reduced the number of pHrodo-labeled puncta after responses to ATP. TRPM5 is known to serve as the downstream effector of the PLC pathway in nonneuronal chemosensory cells, including SCCs, brush cells and tuft cells found in various tissues, and taste receptor cells (Finger and Kinnamon, 2011). TRPM5 activation requires a rapid increase of intracellular Ca^2+^ (Liu and Liman, 2003). Elevated intracellular Ca^2+^ by ATP may subsequently activate TRPM5 channels, further amplifying the Ca^2+^ signal for vesicle release. This likely represents a newly identified role of TRPM5 in these cells.

For potential vesicle release in TRPM5-MCs, Ca^2+^ load may be a key factor, as we found that ATP-responsive TRPM5-MCs showed a higher number of pHrodo-labeled puncta than those cells responsive to the odor mixture. We noticed that the Ca^2+^ responses to ATP lasted longer than the response to odor mixture which presumably increased the total Ca^2+^ load in these cells. Interestingly, we observed spontaneous intracellular Ca^2+^ oscillation in a subset of isolated TRPM5-MCs. When examining pHrodo uptake in these Ca^2+^-oscillating cells, we found that the number of dye-labeled puncta was similar to that found in cells after responses to the odor mixture. These data suggest that the spontaneous oscillation and elevated basal Ca^2+^ levels in these cells were enough for vesicle release without stimulation. In agreement, a recent report showed that tuft cells release basal levels of cytokines, tuning immunity in the gut (von Moltke et al., 2016).

### Endocytosis in the SCs

Using the endocytosis dye pHrodo, we were able to show that both unstimulated and stimulated endocytosis was occurring in the SCs. Unlike the dye labeling in TRPM5-MCs, where we could discern individual pHrodo-labeled puncta, labeling in SCs, especially after activation, was massive, and labeled vesicles or vacuoles were hard to separate. Our pHrodo labeling result is consistent with the positive immunoreaction of EEA1 that labels early endosome in SCs and also consistent with previous electron microscopy findings that SCs contain numerous vesicle and vacuoles, especially in the supranuclear region (Getchell and Mellert, 1991; Getchell and Getchell, 1992). SCs are the key cell type for xenobiotic removal in the upper airway. SCs express a variety of xenobiotic-metabolizing enzymes. Some of these enzymes function intracellularly, which requires engulfing of xenobiotics. Some other enzymes may be secreted to the mucus layer where they metabolize xenobiotics including odor molecules (Menco and Morrison, 2003; Asakawa et al., 2017). Currently, there is little information how these events are regulated. In our study, we found that ACh strongly potentiates dye uptake, and this modification relied primarily on the functional expression of M3-subtype muscarinic receptors. Because of the close anatomical relation between the TRPM5-MCs and SCs, we consider that ACh most likely is released from the cholinergic TRPM5-MCs (Ogura et al., 2011). Release of ACh has been measured in TRPM5-expressing chemosensory cells in the urethra (Deckmann et al., 2014). The cholinergic paracrine regulation would enable TRPM5-MCs to modify and coordinate SC activity with OSNs and potential other MOE cell types in xenobiotic removal and MOE maintenance.

ACh has been found to modulate variety of cellular functions via paracrine pathways. For example, adipose stem cells enhance myoblast proliferation via paracrine secretion of ACh (El-Habta et al., 2018). Granulosa cells and luteal cells in ovary release ACh to promote follicular development and female fertility (Mayerhofer and Fritz, 2002; Urra et al., 2016). In the carotid body, intrinsic release of ACh is used for intercellular coordinated chemical sensing (Kåhlin et al., 2014). In the trachea, ACh from chemosensory brush cells modulates breath rate and airway clearance (Krasteva et al., 2011). Thus, ACh-mediated modification allows different cell types to work in concerted fashion within multicellular networks.

Although we cannot rule out the possibility that ACh from sources other than TRPM5-MCs activates the SCs, our previous study using both transgenic mice and immunolabeling all showed that within the MOE cells, TRPM5-MCs are the only cell type expressing ChAT and VAChT. Their cholinergic nature was further demonstrated by using transgenic ChAT^(BAC)^^−^eGFP mice in which GFP expression is strong in both cell bodies and nerve fibers (Ogura et al., 2011; Krosnowski et al., 2012; Marking et al., 2017). We observed cholinergic fibers (GFP+) in lamina propria innervating submucosal blood vesseles and glandular tissues, but rarely noticed GFP+ nerve fibers penetrating into the cell layers of the MOE. We therefore believe that TRPM5-MCs in the MOE are the primary source for ACh release to regulate SC activity. Future experiments on the release of ACh from TRPM5-MCs will greatly advance our understanding of the MOE cholinergic network and regulation.

### Cholinergic Receptors in SCs

Previously we demonstrated using intracellular Ca^2+^ imaging and immunolabeling that ACh induces Ca^2+^ increases in SCs via muscarinic receptors and expression of M3 and M1 subtypes (Ogura et al., 2011). Our present results obtained using pharmacological agents as well as M3-AChR-KO mice further provide evidence that M3-AChR plays a major role in mediating ACh-induced Ca^2+^ responses in SCs. However, inconsistent results were obtained from the M1-subtype antagonist and agonist. While the M1-selective antagonist pirenzepine did not significantly reduce responses to ACh in control mice, the M1-selective agonist AC-42 induced responses in some SCs. Intriguingly, in SCs isolated from M3-AChR-KO mice we did not observe responses to AC-42. One possible explanation is that AC-42 might have activated the M3 subtype. However, AC-42 reportedly is highly selective for the M1 subtype up to 100 μM in heterologous cells expressing human muscarinic subtypes (Spalding et al., 2002; Jacobson et al., 2010). At maximum levels AC-42 induces phosphatidylinositol turnover and Ca^2+^ mobilization equivalent to 66%–85% of the maximum responses induced by ACh or carbachol (Spalding et al., 2002; Langmead et al., 2006; Jacobson et al., 2010). In our study only a subset of SCs (5 of 43 SCs tested) were responsive to AC-42, and the response amplitude was significantly less than average ACh response amplitude; therefore, M1 receptors, if involved, likely do not play a major role in SCs.

In sum, our results show that TRPM5-MCs dose-dependently respond to ATP and odor mixture and may release ACh to potentiate endocytosis in SCs, possibly promoting xenobiotic removal from the MOE. These results have unveiled cholinergic regulation in the MOE coordinating SC activity important for protecting the epithelium and airway. That TRPM5-MCs are sensitive to ATP and express multiple purinergic receptors also suggests an additional mechanism for the MOE to act in a concerted fashion with the rest of the respiratory mucosa to defend against xenobiotic insults. Taken together, these novel results of cholinergic paracrine signaling in the MOE increase our understanding of how the MOE maintains its function and prevents chemical-induced damage.

## Datasets Are Available on Request

The raw data supporting the conclusions of this manuscript will be made available by the authors, without undue reservation, to any qualified researcher.

## Author Contributions

WLin conceived and supervised the project; wrote the manuscript with drafts and input from the other authors. ZF performed most imaging experiments. TO performed some imaging experiments. WLuo did the immunolabeling. WLin, ZF and TO designed the experiments and analyzed data.

## Conflict of Interest Statement

The authors declare that the research was conducted in the absence of any commercial or financial relationships that could be construed as a potential conflict of interest.

## References

[B1] Asakawa M. Fukutani Y. Savangsuksa A. Noguchi K. Matsunami H. Yohda M. (2017). Modification of the response of olfactory receptors to acetophenone by CYP1a2. Sci. Rep. 7:10167. 10.1038/s41598-017-10862-5 28860658 PMC5579037

[B2] Bezençon C. Fürholz A. Raymond F. Mansourian R. Métairon S. Le Coutre J.. (2008). Murine intestinal cells expressing Trpm5 are mostly brush cells and express markers of neuronal and inflammatory cells. J. Comp. Neurol. 509, 514–525. 10.1002/cne.21768 18537122

[B3] Boase S. Foreman A. Cleland E. Tan L. Melton-Kreft R. Pant H.. (2013). The microbiome of chronic rhinosinusitis: culture, molecular diagnostics and biofilm detection. BMC Infect. Dis. 13:210. 10.1186/1471-2334-13-210 23656607 PMC3654890

[B4] Burnstock G. (2006). Pathophysiology and therapeutic potential of purinergic signaling. Pharmacol. Rev. 58, 58–86. 10.1124/pr.58.1.5 16507883

[B5] Burnstock G. (2007). Purine and pyrimidine receptors. Cell. Mol. Life Sci. 64, 1471–1483. 10.1007/s00018-007-6497-0 17375261 PMC11149472

[B6] Clapp T. R. Medler K. F. Damak S. Margolskee R. F. Kinnamon S. C. (2006). Mouse taste cells with G protein-coupled taste receptors lack voltage-gated calcium channels and SNAP-25. BMC Biol. 4:7. 10.1186/1741-7007-4-7 16573824 PMC1444931

[B7] Coddou C. Yan Z. Obsil T. Huidobro-Toro J. P. Stojilkovic S. S. (2011). Activation and regulation of purinergic P2X receptor channels. Pharmacol. Rev. 63, 641–683. 10.1124/pr.110.003129 21737531 PMC3141880

[B8] Damak S. Rong M. Yasumatsu K. Kokrashvili Z. Pérez C. A. Shigemura N.. (2006). Trpm5 null mice respond to bitter, sweet and umami compounds. Chem. Senses 31, 253–264. 10.1093/chemse/bjj027 16436689

[B9] Deckmann K. Filipski K. Krasteva-Christ G. Fronius M. Althaus M. Rafiq A.. (2014). Bitter triggers acetylcholine release from polymodal urethral chemosensory cells and bladder reflexes. Proc. Natl. Acad. Sci. U S A 111, 8287–8292. 10.1073/pnas.1402436111 24843119 PMC4050540

[B10] Ding X. Dahl A. R. (2003). “Olfactory mucosa: composition, enzymatic localization, and metabolism,” in Handbook of Olfaction and Gustation, 2nd Edn. ed. Doty R. L. (New York, NY: Marcel Dekker, Inc.), 51–73.

[B11] Dunston D. Ashby S. Krosnowski K. Ogura T. Lin W. (2013). An effective manual deboning method to prepare intact mouse nasal tissue with preserved anatomical organization. J. Vis. Exp. 78:e50538. 10.3791/50538 23963491 PMC3854956

[B12] El-Habta R. Kingham P. J. Backman L. J. (2018). Adipose stem cells enhance myoblast proliferation via acetylcholine and extracellular signal-regulated kinase 1/2 signaling. Muscle Nerve 57, 305–311. 10.1002/mus.25741 28686790 PMC5811911

[B13] Farbman A. (2000). “Cell biology of olfactory epithelium,” in The Neurobiology of Taste and Smell, 2nd Edn. eds Finger T. E. Silver W. L. Restrepo D. (New York, NY: Wiley-Liss), 131–158.

[B15] Finger T. E. Böttger B. Hansen A. Anderson K. T. Alimohammadi H. Silver W. L. (2003). Solitary chemoreceptor cells in the nasal cavity serve as sentinels of respiration. Proc. Natl. Acad. Sci. U S A 100, 8981–8986. 10.1073/pnas.1531172100 12857948 PMC166424

[B14] Finger T. E. Kinnamon S. C. (2011). Taste isn’t just for taste buds anymore. Biol. Rep. 3:20. 10.3410/B3-20 21941599 PMC3169900

[B16] Fredholm B. B. Ap I. J. Jacobson K. A. Linden J. Müller C. E. (2011). International union of basic and clinical pharmacology. LXXXI. Nomenclature and classification of adenosine receptors—an update. Pharmacol. Rev. 63, 1–34. 10.1124/pr.110.003285 21303899 PMC3061413

[B17] Gayle S. Burnstock G. (2005). Immunolocalisation of P2X and P2Y nucleotide receptors in the rat nasal mucosa. Cell Tissue Res. 319, 27–36. 10.1007/s00441-004-0979-2 15558320

[B18] Gerbe F. Sidot E. Smyth D. J. Ohmoto M. Matsumoto I. Dardalhon V.. (2016). Intestinal epithelial tuft cells initiate type 2 mucosal immunity to helminth parasites. Nature 529, 226–230. 10.1038/nature16527 26762460 PMC7614903

[B19] Getchell M. L. Getchell T. V. (1992). Fine structural aspects of secretion and extrinsic innervation in the olfactory mucosa. Microsc. Res. Tech. 23, 111–127. 10.1002/jemt.1070230203 1421551

[B20] Getchell M. L. Mellert T. K. (1991). “Olfactory mucus secretion,” in Smell and Taste in Health and Disease, eds Getchell T. V. Doty R. L. Bartoshuk L. Snow J. B. (New York, NY: Raven Press), 83–95.

[B21] Giniatullin R. Nistri A. (2013). Desensitization properties of P2X3 receptors shaping pain signaling. Front. Cell. Neurosci. 7:245. 10.3389/fncel.2013.00245 24367291 PMC3854565

[B22] Gross E. A. Swenberg J. A. Fields S. Popp J. A. (1982). Comparative morphometry of the nasal cavity in rats and mice. J. Anat. 135, 83–88. 7130058 PMC1168130

[B23] Gulbransen B. D. Clapp T. R. Finger T. E. Kinnamon S. C. (2008). Nasal solitary chemoreceptor cell responses to bitter and trigeminal stimulants *in vitro*. J. Neurophysiol. 99, 2929–2937. 10.1152/jn.00066.2008 18417634 PMC2765583

[B200] Hansen A. Finger T. E. (2008). Is TrpM5 a reliable marker for chemosensory cells? Multiple types of microvillous cells in the main olfactory epithelium of mice. BMC Neurosci. 9:115 10.1186/1471-2202-9-115 19055837 PMC2629774

[B24] Hassenklover T. Kurtanska S. Bartoszek I. Junek S. Schild D. Manzini I. (2008). Nucleotide-induced Ca^2+^ signaling in sustentacular supporting cells of the olfactory epithelium. Glia 56, 1614–1624. 10.1002/glia.20714 18551628

[B25] Hausmann R. Kless A. Schmalzing G. (2015). Key sites for P2X receptor function and multimerization: overview of mutagenesis studies on a structural basis. Curr. Med. Chem. 22, 799–818. 10.2174/0929867322666141128163215 25439586 PMC4460280

[B26] Hayakawa Y. Sakitani K. Konishi M. Asfaha S. Niikura R. Tomita H.. (2017). Nerve growth factor promotes gastric tumorigenesis through aberrant cholinergic signaling. Cancer Cell 31, 21–34. 10.1016/j.ccell.2016.11.005 27989802 PMC5225031

[B27] Hayoz S. Jia C. Hegg C. (2012). Mechanisms of constitutive and ATP-evoked ATP release in neonatal mouse olfactory epithelium. BMC Neurosci. 13:53. 10.1186/1471-2202-13-53 22640172 PMC3444318

[B28] Hegg C. C. Greenwood D. Huang W. Han P. Lucero M. T. (2003). Activation of purinergic receptor subtypes modulates odor sensitivity. J. Neurosci. 23, 8291–8301. 12967991 10.1523/JNEUROSCI.23-23-08291.2003 PMC2976511

[B29] Howitt M. R. Lavoie S. Michaud M. Blum A. M. Tran S. V. Weinstock J. V.. (2016). Tuft cells, taste-chemosensory cells, orchestrate parasite type 2 immunity in the gut. Science 351, 1329–1333. 10.1126/science.aaf1648 26847546 PMC5528851

[B30] Hu J. Sheng L. Li L. Zhou X. Xie F. D’Agostino J.. (2014). Essential role of the cytochrome P450 enzyme CYP2A5 in olfactory mucosal toxicity of naphthalene. Drug Metab. Dispos. 42, 23–27. 10.1124/dmd.113.054429 24104196 PMC3876791

[B31] Jacobson M. A. Kreatsoulas C. Pascarella D. M. O’Brien J. A. Sur C. (2010). The M1 muscarinic receptor allosteric agonists AC-42 and 1–[1′-(2-methylbenzyl)-1,4′-bipiperidin-4-yl]-1,3-dihydro-2H-benzimidazol-2-one bind to a unique site distinct from the acetylcholine orthosteric site. Mol. Pharmacol. 78, 648–657. 10.1124/mol.110.065771 20660086

[B32] Jia C. Doherty J. P. Crudgington S. Hegg C. C. (2009). Activation of purinergic receptors induces proliferation and neuronal differentiation in Swiss Webster mouse olfactory epithelium. Neuroscience 163, 120–128. 10.1016/j.neuroscience.2009.06.040 19555741 PMC2728178

[B33] Kåhlin J. Mkrtchian S. Ebberyd A. Hammarstedt-Nordenvall L. Nordlander B. Yoshitake T.. (2014). The human carotid body releases acetylcholine, ATP and cytokines during hypoxia. Exp. Physiol. 99, 1089–1098. 10.1113/expphysiol.2014.078873 24887113

[B34] Kanekar S. Jia C. Hegg C. C. (2009). Purinergic receptor activation evokes neurotrophic factor neuropeptide Y release from neonatal mouse olfactory epithelial slices. J. Neurosci. Res. 87, 1424–1434. 10.1002/jnr.21954 19115410 PMC3097387

[B35] Kawashima K. Fujii T. (2008). Basic and clinical aspects of non-neuronal acetylcholine: overview of non-neuronal cholinergic systems and their biological significance. J. Pharmacol. Sci. 106, 167–173. 10.1254/jphs.fm0070073 18285657

[B36] Krasteva G. Canning B. J. Hartmann P. Veres T. Z. Papadakis T. Muhlfeld C.. (2011). Cholinergic chemosensory cells in the trachea regulate breathing. Proc. Natl. Acad. Sci. U S A 108, 9478–9483. 10.1073/pnas.1019418108 21606356 PMC3111311

[B37] Krosnowski K. Ashby S. Sathyanesan A. Luo W. Ogura T. Lin W. (2012). Diverse populations of intrinsic cholinergic interneurons in the mouse olfactory bulb. Neuroscience 213, 161–178. 10.1016/j.neuroscience.2012.04.024 22525133 PMC3367073

[B38] Kuwabara Y. Alexeeff G. V. Broadwin R. Salmon A. G. (2007). Evaluation and application of the RD50 for determining acceptable exposure levels of airborne sensory irritants for the general public. Environ. Health Perspect. 115, 1609–1616. 10.1289/ehp.9848 18007993 PMC2072859

[B39] Langmead C. J. Fry V. A. Forbes I. T. Branch C. L. Christopoulos A. Wood M. D.. (2006). Probing the molecular mechanism of interaction between 4-*n*-butyl-1–[4-(2-methylphenyl)-4-oxo-1-butyl]-piperidine (AC-42) and the muscarinic M(1) receptor: direct pharmacological evidence that AC-42 is an allosteric agonist. Mol. Pharmacol. 69, 236–246. 10.1124/mol.105.017814 16207821

[B40] Lee R. J. Chen B. Redding K. M. Margolskee R. F. Cohen N. A. (2014). Mouse nasal epithelial innate immune responses to *Pseudomonas aeruginosa* quorum-sensing molecules require taste signaling components. Innate Immun. 20, 606–617. 10.1177/1753425913503386 24045336 PMC4811369

[B41] Lemons K. Fu Z. Aoudé I. Ogura T. Sun J. Chang J.. (2017). Lack of TRPM5-expressing microvillous cells in mouse main olfactory epithelium leads to impaired odor-evoked responses and olfactory-guided behavior in a challenging chemical environment. eNeuro 4:ENEURO.0135-17.2017. 10.1523/eneuro.0135-17.2017 28612045 PMC5467397

[B42] Liman E. R. (2007). “TRPM5 and taste transduction,” in Transient Receptor Potential (TRP) Channels. Handbook of Experimental Pharmacology, (Vol. 179) eds Flockerzi V. Nilius B. (Heidelberg, Berlin: Springer), 287–298.

[B43] Lin W. Ezekwe E. A. Jr. Zhao Z. Liman E. R. Restrepo D. (2008a). TRPM5-expressing microvillous cells in the main olfactory epithelium. BMC Neurosci. 9:114. 10.1186/1471-2202-9-114 19025635 PMC2648980

[B44] Lin W. Ogura T. Margolskee R. F. Finger T. E. Restrepo D. (2008b). TRPM5-expressing solitary chemosensory cells respond to odorous irritants. J. Neurophysiol. 99, 1451–1460. 10.1152/jn.01195.2007 18160424

[B45] Liu D. Liman E. R. (2003). Intracellular Ca^2+^ and the phospholipid PIP2 regulate the taste transduction ion channel TRPM5. Proc. Natl. Acad. Sci. U S A 100, 15160–15165. 10.1073/pnas.2334159100 14657398 PMC299934

[B46] López F. Delgado R. López R. Bacigalupo J. Restrepo D. (2014). Transduction for pheromones in the main olfactory epithelium is mediated by the Ca^2+^-activated channel TRPM5. J. Neurosci. 34, 3268–3278. 10.1523/jneurosci.4903-13.2014 24573286 PMC3935088

[B47] Marking S. Krosnowski K. Ogura T. Lin W. (2017). Dichotomous distribution of putative cholinergic interneurons in mouse accessory olfactory bulb. Front. Neuroanat. 11:10. 10.3389/fnana.2017.00010 28289379 PMC5326757

[B48] Mayerhofer A. Fritz S. (2002). Ovarian acetylcholine and muscarinic receptors: hints of a novel intrinsic ovarian regulatory system. Microsc. Res. Tech. 59, 503–508. 10.1002/jemt.10228 12467026

[B49] Menco B. P. Morrison E. E. (2003). “Morphology of the mammalian olfactory epithelium: form, fine structure, function, and pathology,” in Handbook of Olfaction and Gustation, 2nd Edn. ed. Doty R. L. (New York, NY: Marcel Dekker, Inc.), 17–50.

[B50] North R. A. (2002). Molecular physiology of P2X receptors. Physiol. Rev. 82, 1013–1067. 10.1152/physrev.00015.2002 12270951

[B51] Ogura T. Krosnowski K. Zhang L. Bekkerman M. Lin W. (2010). Chemoreception regulates chemical access to mouse vomeronasal organ: role of solitary chemosensory cells. PLoS One 5:e11924. 10.1371/journal.pone.0011924 20689832 PMC2912856

[B52] Ogura T. Szebenyi S. A. Krosnowski K. Sathyanesan A. Jackson J. Lin W. (2011). Cholinergic microvillous cells in the mouse main olfactory epithelium and effect of acetylcholine on olfactory sensory neurons and supporting cells. J. Neurophysiol. 106, 1274–1287. 10.1152/jn.00186.2011 21676931 PMC3174807

[B53] Rafols J. A. Getchell T. V. (1983). Morphological relations between the receptor neurons, sustentacular cells and Schwann cells in the olfactory mucosa of the salamander. Anat. Rec. 206, 87–101. 10.1002/ar.1092060111 6881554

[B54] Ralevic V. Burnstock G. (1998). Receptors for purines and pyrimidines. Pharmacol. Rev. 50, 413–492. 9755289

[B55] Saunders C. J. Christensen M. Finger T. E. Tizzano M. (2014). Cholinergic neurotransmission links solitary chemosensory cells to nasal inflammation. Proc. Natl. Acad. Sci. U S A 111, 6075–6080. 10.1073/pnas.1402251111 24711432 PMC4000837

[B56] Schütz B. Jurastow I. Bader S. Ringer C. von Engelhardt J. Chubanov V.. (2015). Chemical coding and chemosensory properties of cholinergic brush cells in the mouse gastrointestinal and biliary tract. Front. Physiol. 6:87. 10.3389/fphys.2015.00087 25852573 PMC4371653

[B57] Seminario-Vidal L. Okada S. F. Sesma J. I. Kreda S. M. Van Heusden C. A. Zhu Y.. (2011). Rho signaling regulates pannexin 1-mediated ATP release from airway epithelia. J. Biol. Chem. 286, 26277–26286. 10.1074/jbc.M111.260562 21606493 PMC3143590

[B58] Spalding T. A. Trotter C. Skjaerbaek N. Messier T. L. Currier E. A. Burstein E. S.. (2002). Discovery of an ectopic activation site on the M(1) muscarinic receptor. Mol. Pharmacol. 61, 1297–1302. 10.1124/mol.61.6.1297 12021390

[B59] Stanley A. C. Lacy P. (2010). Pathways for cytokine secretion. Physiology 25, 218–229. 10.1152/physiol.00017.2010 20699468

[B60] Thiebaud N. Sigoillot M. Chevalier J. Artur Y. Heydel J. M. Le Bon A. M. (2010). Effects of typical inducers on olfactory xenobiotic-metabolizing enzyme, transporter and transcription factor expression in rats. Drug Metab. Dispos. 38, 1865–1875. 10.1124/dmd.110.035014 20639433

[B61] Thornton-Manning J. R. Dahl A. R. (1997). Metabolic capacity of nasal tissue interspecies comparisons of xenobiotic-metabolizing enzymes. Mutat. Res. 380, 43–59. 10.1016/S0027-5107(97)00126-7 9385389

[B62] Tizzano M. Gulbransen B. D. Vandenbeuch A. Clapp T. R. Herman J. P. Sibhatu H. M.. (2010). Nasal chemosensory cells use bitter taste signaling to detect irritants and bacterial signals. Proc. Natl. Acad. Sci. U S A 107, 3210–3215. 10.1073/pnas.0911934107 20133764 PMC2840287

[B63] Urra J. Blohberger J. Tiszavari M. Mayerhofer A. Lara H. E. (2016). *In vivo* blockade of acetylcholinesterase increases intraovarian acetylcholine and enhances follicular development and fertility in the rat. Sci. Rep. 6:30129. 10.1038/srep30129 27440195 PMC4954984

[B64] Vogalis F. Hegg C. C. Lucero M. T. (2005). Ionic conductances in sustentacular cells of the mouse olfactory epithelium. J. Physiol. 562, 785–799. 10.1113/jphysiol.2004.079228 15611020 PMC1665525

[B65] von Kügelgen I. (2006). Pharmacological profiles of cloned mammalian P2Y-receptor subtypes. Pharmacol. Ther. 110, 415–432. 10.1016/j.pharmthera.2005.08.014 16257449

[B66] von Moltke J. Ji M. Liang H. E. Locksley R. M. (2016). Tuft-cell-derived IL-25 regulates an intestinal ILC2-epithelial response circuit. Nature 529, 221–225. 10.1038/nature16161 26675736 PMC4830391

[B67] Wakisaka N. Miyasaka N. Koide T. Masuda M. Hiraki-Kajiyama T. Yoshihara Y. (2017). An adenosine receptor for olfaction in fish. Curr. Biol. 27, 1437.e4–1447e.4. 10.1016/j.cub.2017.04.014 28502661

[B68] Wessler I. Kirkpatrick C. J. (2008). Acetylcholine beyond neurons: the non-neuronal cholinergic system in humans. Br. J. Pharmacol. 154, 1558–1571. 10.1038/bjp.2008.185 18500366 PMC2518461

[B69] Workman A. D. Carey R. M. Chen B. Saunders C. J. Marambaud P. Mitchell C. H.. (2017). CALHM1-mediated ATP release and ciliary beat frequency modulation in nasal epithelial cells. Sci. Rep. 7:6687. 10.1038/s41598-017-07221-9 28751666 PMC5532211

[B70] Yamada M. Miyakawa T. Duttaroy A. Yamanaka A. Moriguchi T. Makita R.. (2001). Mice lacking the M3 muscarinic acetylcholine receptor are hypophagic and lean. Nature 410, 207–212. 10.1038/35065604 11242080

[B71] Yamaguchi T. Yamashita J. Ohmoto M. Aoudé I. Ogura T. Luo W.. (2014). Skn-1a/Pou2f3 is required for the generation of Trpm5-expressing microvillous cells in the mouse main olfactory epithelium. BMC Neurosci. 15:13. 10.1186/1471-2202-15-13 24428937 PMC3901341

